# GERiatric Screening in the treatment of elderly patients with Ovarian Carcinoma (GERSOC): study protocol for a pragmatic, cluster randomised controlled trial

**DOI:** 10.1186/s13063-020-4157-y

**Published:** 2020-02-22

**Authors:** Neeltje J. van Soolingen, Carolina H. Smorenburg, Marije E. Hamaker, Wim G. Groen, Valesca P. Retèl, Christianne A. R. Lok, Lonneke V. van de Poll-Franse, Johannes W. Trum

**Affiliations:** 1 Department of Gynaecologic Oncology, Centre for Gynaecologic Oncology Amsterdam, The Netherlands Cancer Institute, Plesmanlaan 121, 1066 CX Amsterdam, The Netherlands; 2 Department of Medical Oncology, The Netherlands Cancer Institute, Plesmanlaan 121, 1066 CX Amsterdam, The Netherlands; 3 Department of Geriatric Medicine, Diakonessenhuis Utrecht, Bosboomstraat 1, 3582 KE Utrecht, The Netherlands; 4 Division of Psychosocial Research and Epidemiology, The Netherlands Cancer Institute, Plesmanlaan 121, 1066 CX Amsterdam, The Netherlands; 5 Department of Health Technology and Services Research (HTSR), University of Twente, Hallenweg 5, 7522 NH Enschede, The Netherlands; 6 CoRPS – Center of Research on Psychology in Somatic diseases, Department of Medical and Clinical Psychology, Tilburg University, Warandelaan 2, 5037 AB Tilburg, The Netherlands; 7 The Netherlands Comprehensive Cancer Organisation, Godebaldkwartier 419, 3511 DT Utrecht, The Netherlands

**Keywords:** Frailty, Elderly, Geriatric screening, Ovarian cancer, Cluster randomisation, Treatment completion, Health-related quality of life, Cost-effectiveness

## Abstract

**Background:**

Approximately 40% of the newly diagnosed patients with advanced ovarian cancer are aged 70 years or older. Standard treatment for advanced disease consists of cytoreductive surgery and combination chemotherapy. In older patients, standard treatment is often withheld or prematurely stopped due to suspected frailty. It remains challenging to distinguish fit elderly patients who can endure standard therapy from frail patients who may benefit from an adapted treatment strategy. As a comprehensive geriatric assessment (CGA) can contribute to the identification of frail patients and improve tailored therapy in this population, screening tests were developed to select those who may benefit from a CGA. However, the use of these geriatric screening tests has rarely been compared with usual clinical care. The GERSOC-trial will evaluate whether geriatric screening in elderly patients with advanced-stage ovarian cancer improves treatment completion and quality of life.

**Methods:**

This pragmatic, cluster randomised controlled trial will be conducted at a minimum of 20 hospitals in the Netherlands. Hospitals are randomly assigned to geriatric screening care (in which a geriatric screening comprised of the G8 questionnaire and the Timed Up and Go test is performed), or care as usual (in which current usual care is continued). A total of 320 patients aged ≥ 70 years with primary, advanced-stage ovarian carcinoma will be included. Patients considered fit on geriatric screening will receive standard treatment; patients who are considered unfit will be referred to a geriatrician for analysis and treatment advice. The primary outcome is the percentage difference in completed standard and adapted therapies between the two study arms. Secondary outcomes include quality of life, cost-effectiveness and survival.

**Discussion:**

This trial aims to gather evidence for the use of geriatric screening in treatment decision-making in elderly patients with advanced ovarian cancer. If proven feasible, beneficial and cost-effective, geriatric screening may be implemented in routine clinical practice.

**Trial registration:**

Netherlands Trial Registry, ID: NL6745. Registered on 2 August 2017.

## Background

In Europe, 35% of the almost 68,000 patients that were newly diagnosed with ovarian cancer in 2018 were aged 70 years and older [1]. In the Netherlands, this applies to almost half of all newly diagnosed patients [2]. The majority of these patients is diagnosed with advanced-stage disease (Fédération Internationale de Gynécologie et d’Obstétrique (FIGO) stage IIB-IV), in which 5-year relative survival rates for patients aged ≥ 70 years are below 20% [3]. Standard treatment of advanced ovarian carcinoma comprises cytoreductive surgery (CRS) and (neo)adjuvant chemotherapy consisting of carboplatin and paclitaxel [4, 5].

Although almost half of the newly diagnosed patients are elderly, this population is underrepresented in randomised clinical trials investigating the optimal treatment of advanced ovarian carcinoma. In addition, conventional endpoints for clinical studies are not always suitable for elderly cancer patients, as comorbidities may influence survival and patients may prioritise quality of life over increased survival [6]. Therefore, it is uncertain to which extent the current evidence for ovarian cancer treatment can be extrapolated to the elderly population. As a result, it remains unclear which patients can endure the burdensome standard therapy and for whom treatment should be adapted.

Several observational studies have shown that elderly ovarian carcinoma patients receive standard treatment less frequently than their younger counterparts. This applies to both chemotherapy and CRS [3, 7–11]. Older patients less often have complete surgery [10] and suffer more frequently from postoperative complications and mortality [12–14]. A Dutch single-centre cohort study [15] found that 11 out of 47 patients (23%) aged 70 years or older who were considered fit for standard treatment, were not able to complete treatment without adjustments. These findings reflect the difficulties in predicting whether an elderly woman with advanced ovarian cancer is too frail to tolerate the standard treatment.

Recognising frailty is of major importance in improving treatment selection in elderly ovarian cancer patients. Frail patients experience an accelerated decline in physiological reserves, leading to an increased vulnerability to unfavourable outcomes (such as delirium and falls) following stressors [16]. In addition, in the sight of the increasing financial burden of hospital care, improved selection for the right treatment will be beneficial for society as well. A comprehensive geriatric assessment (CGA) evaluates frailty at a multidimensional level across physical, functional and psychosocial domains and may be adapted for use in oncology. Its main goal is to identify targets for geriatric interventions and to guide selection of a tailored treatment strategy [17].

As not every patient needs an extensive geriatric evaluation, unnecessary referral to a geriatrician should be avoided. Therefore, various geriatric screening tools have been developed to identify patients who may benefit from a CGA. Currently, no specific screening tool is recommended for standard use in oncology [18]. Two validated and commonly used screening tools are the G8 questionnaire and the Timed Up and Go test (TUG). The G8 questionnaire is developed specifically for geriatric-oncologic patients and covers multiple domains of frailty [19, 20]. The TUG assesses mobility in a short walking test [21] and is associated with survival and treatment-related complications [22]. Although multiple studies have assessed the influence of a geriatric evaluation on treatment decisions, few have focused on the actual clinical benefit of treatment decisions guided by any form of geriatric evaluation. Consequently, it remains unclear whether the use of a geriatric screening in clinical practice will indeed improve treatment outcomes.

The aim of this trial is to establish the use of a geriatric screening and subsequent CGA in treatment decision-making for elderly women with advanced ovarian cancer. We aim to investigate whether the introduction of a geriatric screening tool as compared to care as usual improves treatment completion, leading to better quality of life in a cost-effective way in this vulnerable population.

## Methods

### Trial design and setting

**Fig. 1:**
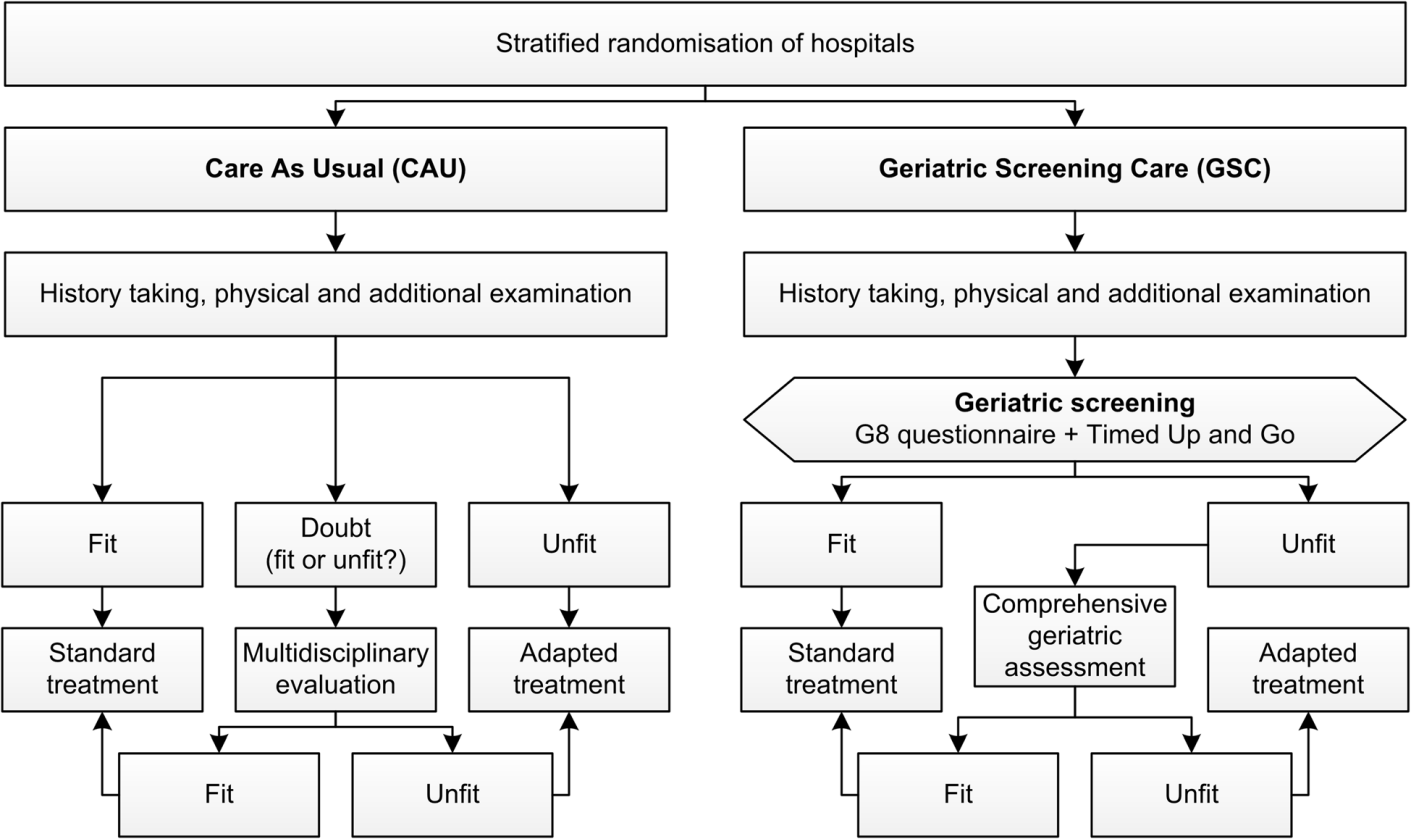
Study design of the GERSOC-trial

**Fig. 2:**
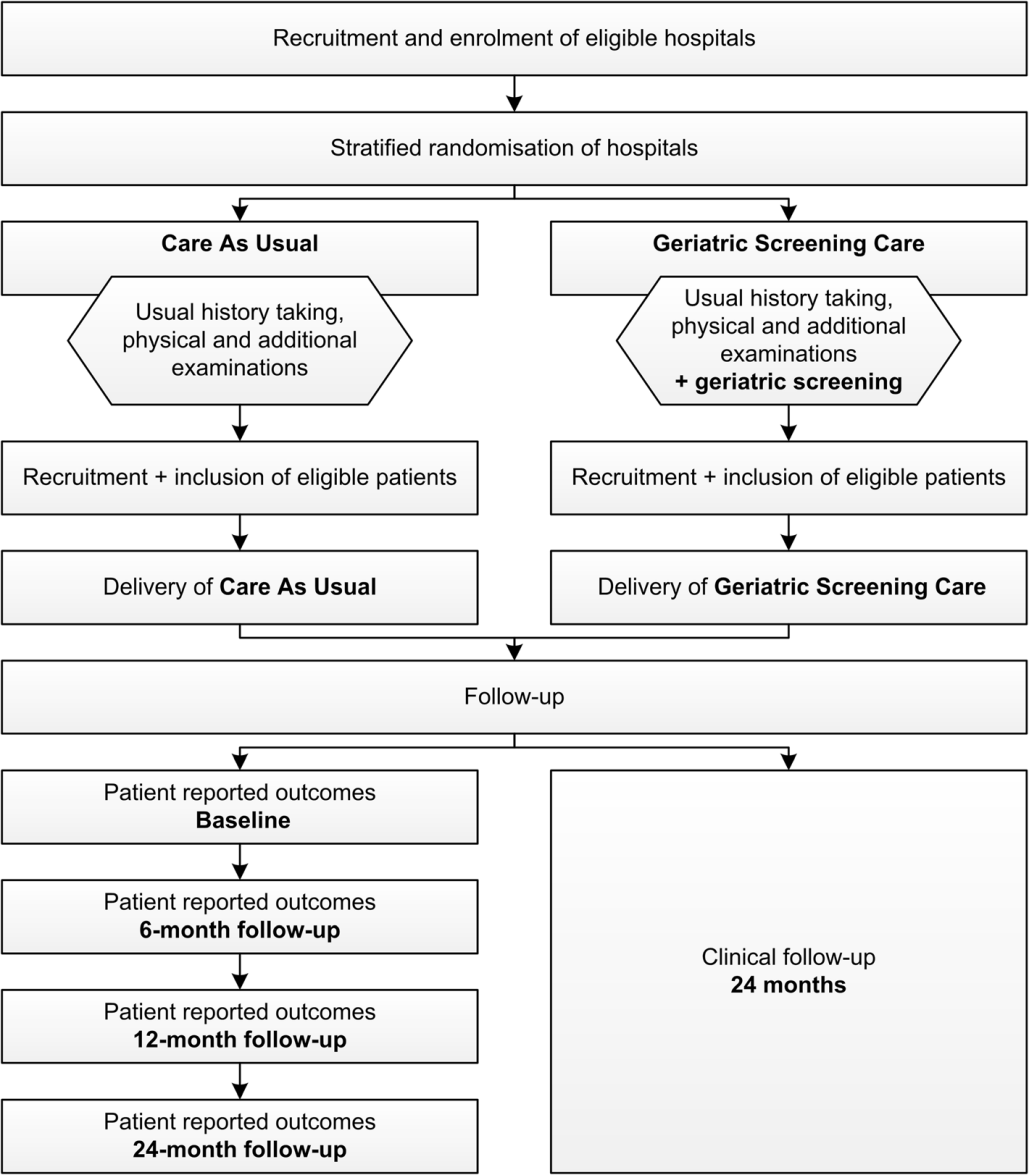
Schematic outline of study procedures in the GERSOC-trial

The GERSOC-trial is a pragmatic, cluster randomised controlled trial that will be performed at a minimum of 20 university and non-university hospitals throughout the Netherlands. Participating centres will be randomised 1:1 to either Geriatric Screening Care (GSC) or Care As Usual (CAU). A total of 320 patients with newly diagnosed advanced ovarian cancer will be included in this prospective trial. The use of a geriatric screening to decide whether or not to refer a patient to the geriatrician will be compared to usual care. Schematic outlines of the study design and the study procedures are shown in Figs. 1 and 2. The study protocol is presented using the Standard Protocol Items: Recommendations for Interventional Trials (SPIRIT) Checklist in Additional file 1.

### Objectives

The aim of the study is to demonstrate the impact of a geriatric screening test prior to treatment decision and start of therapy on treatment completion rates in ovarian cancer patients aged 70 years or older. Secondary outcomes include progression-free survival (PFS), overall survival (OS) and disease-specific survival (DSS); health-related quality of life (HRQL); cost-effectiveness; and toxicity of treatment. Also, we aim to gain insight into the feasibility of the geriatric screening tests in daily practice and its impact on changes in supportive measures and treatment in the study population.

Geriatric screening is thought to improve the selection of frail patient who require a CGA, which may result in a more tailored treatment strategy. Therefore, we hypothesise that standard as well as adapted treatment is completed more often by patients who receive GSC than by those who receive CAU. Furthermore, we hypothesise that patients in the GSC arm will report less toxicity and better HRQL than patients with CAU. Finally, we expect that the introduction of a geriatric screening in this population is a cost-effective intervention.

### Study population

#### Inclusion and exclusion criteria

Patients aged 70 years or older who are diagnosed with primary ovarian carcinoma (including fallopian tube and peritoneal carcinoma) FIGO stages II, III or IV are eligible to participate in the study. Patients with a high suspicion of advanced-stage ovarian cancer, in whom diagnosis is not yet pathologically confirmed before primary CRS, can also be included. A high suspicion of advanced ovarian cancer should then be based on imaging (i.e. signs of pelvic, abdominal or extra-abdominal metastases). Patients must be able to complete a Dutch questionnaire and written informed consent has to be obtained before inclusion in the trial. Patients who cannot read or write Dutch will be excluded from participation in the study, as they will not be able to complete a Dutch questionnaire.

#### Withdrawal and replacement

Patients can refuse to participate or leave the study at any time, without any consequences. In case a patient chooses to withdraw from the study, the collected data will still be used for the study as described in the patient information leaflet and signed informed consent form. Patients with a high suspicion of advanced-stage ovarian cancer who are included prior to surgery are withdrawn from the study when the diagnosis of advanced ovarian cancer is not confirmed by pathology after surgery. Replacement of withdrawn patients will only occur if the aforementioned histopathological criteria for withdrawal are met.

### Randomisation, recruitment and allocation

#### Randomisation

Cluster randomisation was considered the most suitable randomisation method for this study for two reasons. First, we believe that individual randomisation is not appropriate because health care providers then have to alternate CAU and GSC between patients. By performing geriatric screening in patients randomised to GSC, the health care giver will become more aware of possible geriatric risk factors and may subsequently recognise these factors in patients randomised to CAU. It is very likely that such a geriatric screening approach will influence usual care and thus study results. Second, when patients randomised to CAU become aware that other patients were randomised to GSC and receive geriatric screening tests, they may become dissatisfied with their usual care. This could lead to biased results on quality of life.

In our trial, clusters consist of hospitals involved in diagnosis and treatment of ovarian cancer. These hospitals will be randomised by the coordinating study centre after local approval is obtained. Randomisation to either CAU or GSC will be performed in the web-based randomisation software ALEA (version 17.1) using minimisation (with a 75% probability that the underrepresented arm is selected in case of imbalance).

In the Netherlands, CRS for ovarian cancer is centralised in gynaecological oncology centres. It is possible that these centres will include more patients than hospitals that do not perform CRS. To prevent imbalance between the clusters, we will perform a stratified randomisation based on gynaecological oncology centre, defined as a hospital that performs at least 20 CRS procedures for primary ovarian cancer per year.

Following the nature of the intervention, allocation of the hospitals will not be blinded. The Medical Research Ethics Committee (MREC) determined that the patients allocated to GSC must be informed about randomisation on hospital level. Due to these restrictions, the geriatric screening intervention will not be introduced as ‘standard care’ for all patients in hospitals randomised to GSC, but will only be performed in patients who have signed informed consent.

#### Recruitment and allocation

Patients will be recruited by their gynaecologic oncologist, medical oncologist or nurse (practitioner). Allocation is based on the hospital where the patient is treated. Eligible patients receive a short briefing and a leaflet with information about the study. The patient information leaflet in CAU will be generic, stating that we investigate the quality of life of older ovarian carcinoma patients. The patient information leaflet in GSC will contain information about the use of geriatric screening and possible referral to a geriatrician. After obtaining written informed consent, participating patients will be registered in ALEA and subsequently assigned a unique study number for further data handling. Eligible patients who do not want to participate in the study will also be registered, including the reason for refusal.

### Intervention versus usual care

#### Care as usual

When the hospital is randomised to CAU, medical care will be provided according to the current guidelines [4]. As the Dutch guidelines for ovarian cancer do not provide any specific advices for care of the elderly patient, decisions of standard or adjusted treatment are based on the treating physicians opinion and multidisciplinary consultation. In general, the final treatment plan will be defined in the multidisciplinary team meeting. If considered necessary, patients may be referred to other specialists for further evaluation at the discretion of the treating physician. Implementing CAU will not alter the current clinical pathways, as usual care in the participating hospital will be continued and no intervention is introduced. All concomitant medication and care within and outside treatment for ovarian cancer is allowed.

#### Geriatric screening care

In hospitals randomised to GSC, all participating patients will receive a geriatric screening after (suspected) diagnosis of advanced ovarian cancer. The geriatric screening consists of the G8 questionnaire and the TUG [19, 21]. Patients determined unfit on either or both of the tests will be referred to a geriatrician for CGA. The geriatric screening as well as the CGA will be performed before a treatment strategy is defined. At the start of the study, the involved health care professionals will receive a brief instruction on the execution of the geriatric screening. Deliberate deviation from the treatment advice is permitted if the treating physician considers this necessary. Implementing GSC will alter the current clinical pathway of patients who participate in the study, as they will receive a geriatric screening and will possibly be referred to a geriatrician for additional treatment advice. All concomitant medication and care within and outside treatment for ovarian cancer is allowed.

##### G8 questionnaire

The G8 questionnaire consists of eight questions addressing age, food intake, weight, mobility, neuropsychological problems, body mass index, medication use and self-perceived health. The final score ranges from 0 (heavily impaired) to 17 (not at all impaired) and the cut-off value is 14 [19]. Patients with a final score of ≤ 14 will be referred for CGA.

##### Timed Up and Go test (TUG)

The TUG [21] assesses gait speed, walking pattern and balance in older patients. To perform the TUG, patients are asked to stand up from a chair with armrests, walk three metres, turn around, return to the chair and sit down again. The time to complete this procedure is recorded. After one training round, the test is repeated three times and the average duration is calculated. Patients with a TUG > 20 s are considered frail and need further evaluation by a geriatrician.

##### Definition of fit and unfit patients

Patients considered fit based on predefined outcomes on both of the tests (G8 questionnaire > 14 points and TUG ≤ 20 s) will receive standard treatment. Patients considered unfit by the G8 questionnaire (≤ 14 points) and/or TUG (> 20 s) will be referred to a geriatrician for CGA.

##### Comprehensive geriatric assessment for unfit patients

The geriatrician will perform a CGA in accordance with the guidelines of the Dutch Geriatrics Society (NVKG) [23]. This will result in a description of the patient’s health status across somatic, psychological, functional and social domains and report presence of any of the following: new or insufficiently treated comorbidity, polypharmacy, cognitive impairment, mood disorders, (risk of) malnutrition, impaired mobility, dependence in basic and/or instrumental activities of daily living and social support. Wherever possible, the geriatrician will initiate interventions to minimise the impact of these impairments and to optimise the patient's quality of life and ability to tolerate treatment. Subsequently, the geriatrician will communicate these interventions and considerations on treatment adjustments with the treating physician. The geriatrician’s advice will be incorporated into a tailored therapy proposal for each patient.

### Outcome measures

The primary outcome is the percentage of patients who started and completed standard or adapted treatment. Completed standard treatment is defined as a dose intensity of chemotherapy of ≥ 75%, CRS within six weeks after the last neoadjuvant chemotherapy course and start of adjuvant chemotherapy within six weeks after CRS. Adjustments of standard treatment may include: change in chemotherapy regimen (such as carboplatin monotherapy), omit CRS, or refrain from active treatment (best supportive care only). Completion of adapted treatment is described as completion of the treatment plan as defined prior to start of treatment with a dose intensity of chemotherapy of ≥ 75%.

Secondary outcome parameters include toxicity of treatment; PFS, OS and DSS; HRQL; primary and secondary treatment adjustments; incremental costs; incremental effect in terms of Quality Adjusted Life Years (QALY’s); and incremental cost-effectiveness.

### Data collection

#### Clinical outcomes

Clinical, demographic and socio-economic data will be collected from medical charts. In addition to basic clinical data, we will focus on comorbidity, geriatric risk factors and geriatric assessment. These data will be collected in a web-based electronic case report form (ALEA, version 17.1) and will be stored in coded form using the unique study number. Clinical data will be collected up to two years of follow-up.

#### Patient-reported outcomes

Cancer-specific HRQL will be measured using the EORTC QLQ-C30 questionnaire [24], designed and validated specifically to measure quality of life in cancer patients. It comprises functional and symptom scales, a scale on global health and quality of life and several single item symptoms. This core instrument will be complemented by the ovarian cancer specific module EORTC QLQ-OV28 [25]. This questionnaire focusses on symptoms and side effects specific for ovarian cancer.

For the cost-effectiveness analysis, utilities will be retrieved to derive QALY’s by means of the EuroQol-5D-5L (EQ-5D-5L) questionnaire [26]. Utilities reflect the preferences of society for length of life corrected for the quality of those life years. Direct healthcare cost data will be collected from the hospitals administrative database. Indirect costs data will be derived bottom-up from participating health care providers and using an abbreviated version of the institute for Medical Technology Assessment’s Medical Consumption Questionnaire for costs outside the hospital (iMCQ) [27]. Patients are also asked to report additional demographic and socio-economic data.

All patient-reported outcomes will be collected before start of treatment and 6, 12 and 24 months after diagnosis. Patients will receive the first questionnaire from the nurse or treating physician. Patients will return the completed questionnaire in a pre-stamped envelope to the coordinating study centre. The subsequent questionnaires will be sent to the patient by the central study coordinator and can be completed on paper or online after secured login (www.profielstudie.nl). Non-respondents will be sent a reminder letter and questionnaire by mail or e-mail after four weeks.

### Timeline

**Fig. 3:**
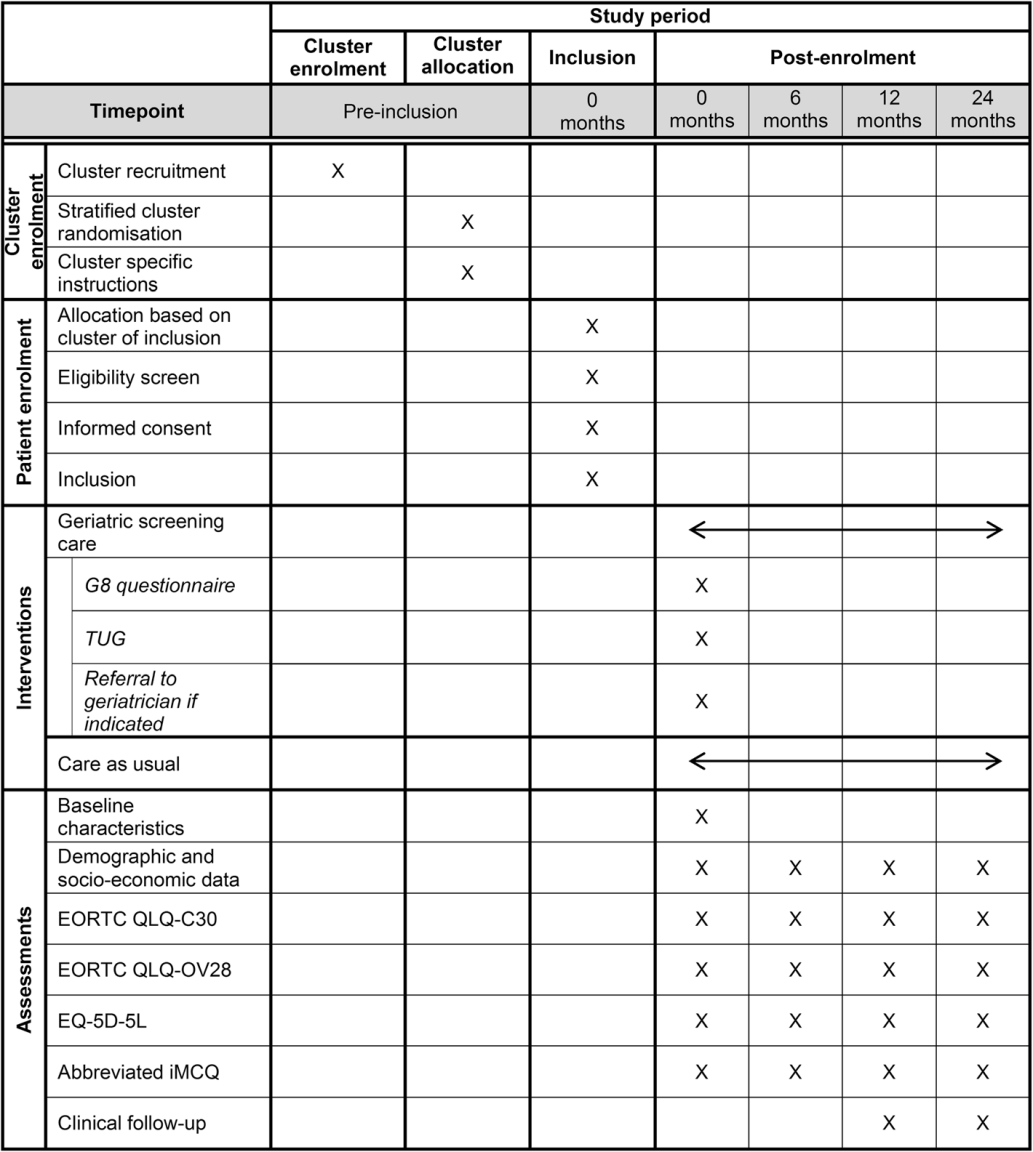
SPIRIT figure: overview of enrolment, interventions and assessments in the GERSOC-trial

The SPIRIT figure (Fig. 3) represents a complete schedule on study activities in the GERSOC-trial.

### Sample size calculation

The primary outcome is defined as the percentage of patients who started and completed treatment in both study arms. A previous study has shown that approximately 75% of the patients aged 70 years and over with advanced stage ovarian cancer was able to complete standard treatment, implying a failure rate of 25% to complete optimal treatment [15]. To demonstrate a 13% point difference from 25% in the control group of standard care to 12% in the intervention group, with a power of 80% and a two-sided statistical significance (alpha) level of 5%, a sample of circa 272 patients would have been necessary when we would have conducted an individually randomised controlled trial. However, in cluster randomised trials observations on participants within a single cluster tend to be correlated, leading to a lower effective sample size than the total number of individual participants. As a result, cluster randomised trials demand more participants than individually randomised trials to acquire similar statistical power [28].

The intracluster correlation coefficient (ICC) compares the within group variance with the between group variance and represents the similarity among subjects within clusters. ICC ranges from 0 to 0.1 are considered common. In a previous study among gynaecological cancer patients a very low ICC (< 0.01) was observed for HRQL measures. This suggests that the within cluster variance is much greater than the between cluster variance. Additionally, stratified randomisation will be applied to further reduce between-cluster variability.

The corresponding number of patients that need to be included per cluster (m) is calculated with the ICC (?), the number of clusters (k) and the required sample size in an individually randomised trial (nl) in the equation: m = nl (1 - ?) / (k – nl?). Taking into account a minimum number of 20 clusters (participating hospitals) in our trial, this translates to m = 272 (1–0.01) / (20–272 * 0.01) = 16. Thus, we need to include 16 individuals per cluster, leading to an overall sample size of 20 * 16 = 320 patients.

### Statistical analysis

All statistical tests will be two-sided and considered significant if *p* < 0.05. We will use cluster specific analyses because the geriatric screening intervention is targeted at the hospital level and the effects will be evaluated for individual patients. We will perform a descriptive statistical analysis of organisational and socio-demographic characteristics at baseline in order to assure the comparability of the intervention and control group. Baseline measures and changes in outcome variables in time for each study-arm will be presented as means (± SD). All analyses will be adjusted for baseline and hospital variables that differ significantly between groups.

#### Clinical outcomes

The primary outcome ‘percentage of completed standard and adapted treatment’ will be compared between the intervention and control group. Comparisons of the percentages will be analysed using logistic regression, adjusting for differences in patient and hospital characteristics at baseline. Secondary outcomes, such as treatment adjustments during follow-up, will be compared between the two groups using logistic regression analyses. PFS, OS and DSS will be estimated using the Kaplan-Meier method. Conventional survival analyses do not allow us to account for the loss of independence that potentially follows from clustering of patients within one centre. Therefore, differences between the groups will be compared using Cox regression models with mixed effects: this approach modifies a Cox proportional hazards regression model by incorporating cluster-specific random effects that modify the baseline hazard function [29].

#### Health-related quality of life and toxicity

We will use a multilevel modelling approach [30] to investigate differences between the intervention and control group. This approach is appropriate to account for the clustering at hospital level [31]. Repeated measures analysis using generalized estimating equations will be carried out to account for the intra-patient dependency of the repeated measures [32]. In addition, differences in effect of the geriatric screening and usual care at the different time points will be evaluated [32]. Missing outcomes will be assumed missing at random. An advantage of the use of a multilevel modelling approach is that all patients can be included in the analyses, regardless any missing follow-up measurements. Clinically meaningful differences in HRQL scores will be based on previously published medium/large minimally important deteriorations in the EORTC QLQ-C30 scales [33]. Data on patient and tumour characteristics requested from the Netherlands Cancer Registry will be used to compare the group of respondents, non-respondents and patients with unverifiable addresses, using t-tests for continuous variables and Chi-square analyses for categorical variables.

#### Health Technology Assessment

Health Technology Assessment (HTA) consists of two parts: First, a scenario analysis to anticipate on barriers and facilitators for implementation will be effectuated. This analysis consists consecutively of the identification of (dynamic) aspects having impact on adoption; a brainstorm on possible scenarios by informal interviews with experts; a scenario construction; validation of the scenarios by means of semi-structured interviews with experts and finally, a quantification into parameters for cost-effectiveness modelling. A cost-effectiveness analysis (CEA) will be performed, with incremental costs per QALY gained as most important outcome.

For the CEA, a Markov model will be constructed with four mutually exclusive health states: disease free survival, recurrence, distant metastasis and death. Using a monthly cycle length, the model will simulate the course of events in a hypothetical cohort of 1000 patients with an average age of 75 years and stage III ovarian cancer. A societal and hospital perspective, plus lifelong time horizon will be adopted. The gathered direct and indirect costs from the trial will be included. Subsequently, costs for treatment and treatment of recurrences, follow-up and palliative care will also preferably be based on the trial, but in case of insufficient numbers, these will be based on literature.

Mostly, trial data on costs are not normally distributed, therefore the non-parametric Mann-Whitney U-test will be applied, with a two-sided significance level α = 0.05. Differences in treatment arms will be tested using the Student's t-test; the paired t-test will be used for differences in time.

Incremental cost-effectiveness ratios will be calculated and stochastic parameter uncertainty handled probabilistically (10,000 Monte Carlo simulations). Distributions will be assigned to the parameters. According to the Dutch guideline [34], future costs and effects will be discounted to their present value by a rate of 4% and 1.5% per year respectively. The cost-effectiveness model will be tested using (deterministic) sensitivity analyses. The results of the probabilistic sensitivity analysis will be illustrated in a cost-effectiveness plane, and decision uncertainty will be shown by cost-effectiveness acceptability curves [35]. The latter will show the probability that a pathway has the highest net monetary benefit, and thus is assumed cost-effective, for a range of Willingness to Pay values for one additional QALY (the ceiling ratio). In this analysis, we will use the Dutch (informal) ceiling ratio of € 80,000 per QALY [36]. A budget impact analysis will be performed following the ISPOR guidelines [37]. We will use hospital perspective and a 5-year time horizon. For all other parameters, the input parameters of the CEA will be used.

### Trial management

#### Roles, responsibilities and auditing trial conduct

The project management group from the coordinating centre (The Netherlands Cancer Institute) will include and randomise participating hospitals, assure (local) ethical approval, initiate the study at all sites, provide instruction on geriatric screening, address questions concerning the trial, collect the data, complete follow-up and analyse the data. A list of study sites can be obtained from the coordinating centre as well. The steering committee will oversee inclusion rates, data collection and data analysis and propose interventions if necessary. The project management group will meet every month to review trial conduct and the steering committee will meet once a year. A report on the progress of the trial will be submitted to the accredited MREC once a year.

#### Data monitoring and interim analysis

Concerning the need for a Data Monitoring Committee (DMC), the Dutch Central Committee on Research Involving Human Subjects (CCMO) refers to the European Medicines Agency (EMA) guideline on data monitoring committees [38]. Although advanced stage ovarian cancer is a life threatening disease, the intervention in this trial does not concern a pharmaceutical product or device, is well characterised and not considered being potentially harmful to patients. The G8 questionnaire and the TUG are validated and commonly used for the identification of frail elderly patients. The intervention is not blinded and is performed only once per patient. Consequently, this trial does not include a DMC. The trial protocol does not describe standardised reporting on (Serious) Adverse Events ((S)AEs), as we do not expect to that participation in the study or receiving a geriatric screening will result in any (S)AEs. However, if unforeseen and unintended adverse effects occur and are spontaneously reported to the study team, these will be assessed and reported to the MREC if necessary. In the absence of a DMC and considering the trial is unblinded and that no safety issues are expected, no interim analyses or formal stopping rules are established. The absence of interim analysis, standardized (S)AE reporting and a DMC in this trial is approved by the responsible MREC.

### Ethical considerations and data security

This trial was approved by the accredited MREC of The Netherlands Cancer Institute – Antoni van Leeuwenhoek (MREC AVL) for the original protocol and all amendments. Protocol amendments will be communicated to all relevant parties. The study will be conducted in accordance with the Declaration of Helsinki, the Guidelines for Good Clinical Practice, the Dutch Act on Medical Research Involving Human Subjects (WMO) and the Dutch law in general. A WMO subject insurance is provided for all participants.

Confidentiality will be guaranteed with the assignation of a study number to each participant. Returned questionnaires have no names attached and will be linked to clinical data by study number. All data will be stored for 15 years according to the current WMO guidelines. The handling of personal data will comply with the EU General Data Protection Regulation and the Dutch Act on Implementation of the General Data Protection Regulation.

The patient information leaflet describes the people and regulatory authorities that may possibly get access to the (uncoded) study data and the reason why these people or authorities may need access to these data. On the informed consent form, participants are asked to sign for permission for the use of their data as described in the patient information leaflet.

The results of the trial will be published in scientific journals, presented at (inter)national conferences and disclosed to patients via patient organisations.

## Discussion

By introducing a geriatric screening in the work-up for treatment of elderly patients with advanced ovarian carcinoma, the GERSOC-trial aims to increase treatment completion rates and quality of life in this vulnerable population.

To our knowledge, this is the first nationwide pragmatic cluster randomised trial evaluating the effect of geriatric screening on clinical outcomes for ovarian cancer patients ≥ 70 years of age. As the elderly population is rapidly increasing, the need for tools to distinguish patients who are fit for extensive oncologic treatment from those who will benefit from adapted treatment strategies is emerging. The introduction of a geriatric screening may be a relatively simple but effective way to enable gynaecologists and oncologists to improve patient-tailored care. However, it is still uncertain whether the use of a geriatric screening tool to identify frail patients, will lead to better clinical and patient reported outcomes. If geriatric screening is not superior to the physicians judgment, performing this screening and referral to a geriatrician cannot be considered cost-effective. Therefore, we have to evaluate how often geriatric screening will lead to referral to a geriatrician and should carefully consider how much this affects the workload of geriatricians. If geriatric screening in older patients with ovarian cancer proves to be beneficial, it should be implemented in routine clinical practice. As referral to a geriatrician is covered by the health insurance, this will be no barrier to implementation.

Some issues may impact the execution of the trial. As ovarian cancer has a relatively low incidence rate and elderly patients tend to participate less frequently in clinical trials, individual physicians have to be extra alert to recall the study and invest time to facilitate inclusion of eligible patients. Additionally, the MREC demanded to inform patients in GSC about the randomisation on hospital level. As a result, signed informed consent has to be obtained before geriatric screening is performed. This may lead to a narrow window for inclusion, a higher number of refusing patients, and lagging accrual in the intervention trial-arm. Finally, the pragmatic design of this trial facilitates easy implementation of the geriatric screening in clinical care, but also allows variations in execution of the screening between sites. To anticipate the issues mentioned above, close contact will be held with local investigators and inclusion rates will be strictly monitored.

This trial aims to gather evidence for the use of geriatric screening in treatment decision-making in elderly patients with advanced ovarian cancer. If proven feasible, beneficial and cost-effective, geriatric screening may be implemented in routine clinical practice.

## Trial status

At present, hospital and patient recruitment for this trial is ongoing. At the time of submission, twenty hospitals are participating in the trial. The first patient was included in July 2018 and recruitment is expected to be completed in August 2021. The original protocol (version 1.2, dated November 23, 2017) has been approved by the MREC AVL at December 14, 2017. The most recent approved version of the protocol is version 1.4, dated March 19, 2019.

## Supplementary information

**Additional file 1.** Standard Protocol Items: Recommendations for Interventional Trials (SPIRIT) checklist. SPIRIT checklist applying to the study protocol article “GERiatric Screening in the treatment of elderly patients with Ovarian Carcinoma (GERSOC): study protocol for a pragmatic, cluster randomised controlled trial”.

## Data Availability

Clinical and socioeconomic data collected in ALEA will be coded and remain property of The Netherlands Cancer Institute. Any party can contact the institute or principal investigator with a request of a license on the data, which will be judged on scientific relevance and privacy aspects by the data access board of the institute. Any such request however will not be unreasonably withheld. For each participating centre, clinical data of its own patients will continuously be available for the treating physician and only in the patients interest. The HRQL data will be collected within the PROFILES registry (www.profilesregistry.nl). The PROFILES registry provides an (inter)national resource for research into the patient (reported) outcomes after cancer. PROFILES data is open access available for non-commercial scientific research, subject only to privacy and confidentiality restrictions and only after publication of the present project.

## Abbreviations

CAU: Care As Usual
CCMO: Central Committee on Research Involving Human Subjects
CEA: Cost-Effectiveness Analysis
CGA: Comprehensive Geriatric Assessment
CRS: Cytoreductive surgery
DMC: Data Monitoring Committee
DSS: Disease Specific Survival
EMA: European Medicines Agency
EQ-5D-5L: EuroQol-5D-5L questionnaire
FIGO: Fédération Internationale de Gynécologie et d’Obstétrique
GSC: Geriatric Screening Care
HRQL: Health-Related Quality of Life
HTA: Health Technology Assessment
ICC: Intracluster Correlation Coefficient
iMCQ: iMTA (institute for Medical Technology Assessment) Medical Consumption Questionnaire
MREC: Medical Research Ethics Committee
NVKG: Dutch Geriatrics Society (in Dutch: Nederlandse Vereniging voor Klinische Geriatrie)
OS: Overall Survival
PFS: Progression-Free Survival
(S)AE: (Serious) Adverse Event
TUG: Timed Up and Go test
QALY: Quality Adjusted Life Year
WMO: Dutch Act on Medical Research Involving Human Subjects
ZonMw: The Netherlands Organisation for Health Research and Development
